# London Mortality Tables for the Quarter Ending September 26

**Published:** 1841-01

**Authors:** 


					1841.] 267
PART FIFTH.
JWefctcal 3ntelUg;etue?
TABLE OF MORTALITY FOR LONDON,
For the Third Quarter of 1840:
Showing the number of Deaths from all causes registered in thirteen weeks, from the
4th July to the 2($th September, 1840.
Causes of Death.
Class r.
Smallpox
Measles
Scarlatina
Hooping-cough
Croup
Thrush
Diarrhoea
Dysentery
Cholera
Influenza
Typhus
Erysipelas
Syphilis
Hydrophobia
Total Epidemic, &c..
Class ir.
Cephalitis
Hydrocephalus
Apoplexy
Paralysis
Convulsions
Epilepsy
Insanity
Delirium tremens
Diseases of the brain, &c...
Total Dis. of Brain, &c.
Class nr.
Quinsey
Bronchitis
Pleurisy
Pneumonia
Hydrothorax
Asthma
Consumption
Diseases of the lungs, &c..
Total Dis. of Lungs, &c.
Class iv.
Pericarditis
Aneurism
Diseases of the heart, &c...
Total Dis. of Heart, &c.
Week ending
4th Ilth 18th 25th
157
4
7
3
44
0
9
156
5
14
18
179
August.
Week ending
1st 8th 15th 22d 29th
1
7
1
51
3
9
146
6
224
163
14
157
216 212
1 1
0| 2
18 9
19, 12
Skptembkr.
Week ending
5th 12th 19th 26th
12 14
172
29i 26
2
207
239
299
520
198
86
123
257
26
48
15
3 a
74
1
163
467
198
200
834
45
10
19
109
22
68
21
590
48
123
1805
120
2797
12
12
200
268 Medical Intelligence. [Jan.
Causes of Death.
Class v.
Teething
Gastritis, enteritis ....
Peritonitis
Tabes mesenterica ...
Ascites
Ulceration
Hernia
Colic or ileus
Diseases of the stomach,&c.
Hepatitis
Jaundice
Disease of the liver, &c. ..
Total Dis. of Stomach, &c.
Class vi.
Nephritis
Diabetes
Stone
Stricture
Diseases of the kidneys,&c.
Total Dis. of Kidneys, &c.
Class vri.
Childbed
Ovarian dropsy
Diseases of uterus, &c
Total Dis. of Uterus, &c.
Class viii.
Rheumatism
Diseases of joints, &c
Total Dis. of Joints, &c.
Classix.
Ulcer
Fistula
Diseases of skin, &c
Total Dis. of Skin, &c.
Class x.
Inflammation
Hemorrhage
Dropsy
Abscess
Mortification
Scrofula
Carcinoma
Tumour
Gout
Atrophy
Debility
Malformations
Sudden deaths
Total Dis. of Uncertain Seat.
Class xi.
Old age or natural decay ..
Class xn.
Intemperance
Privation
Violent deaths
Total by Violence, &c.
Class xm.
Causes not specified ....
Total Deaths from all causes
July.
Week ending
4th 11th 18th 25th
22
26
76
12
94
831 840 867 851 829! 848
August. I September.
Week ending I Week ending
1st 8th 15th 22d 29th 5th 12th 19th 26th
108
46
92 90
27
56
13
20
97
36
63
17
Total.
875 820 857 853 821 838 11008
1841.] Dr. Carswell. 269
NUMBER OF DEATHS IN THE DIFFERENT DISTRICTS.
Districts.
Estimated Population
in 1840.
Deaths during
the Quarter.
West Districts
North Districts
Central Districts
East Districts
South Districts
308,921
414,458
369,722
411,634
450,265
1602
2087
2121
2441
2757
^Total (Males 5714, Females 5294)
1,955,000
11,008

				

